# The digital age in retinal practice

**DOI:** 10.1186/s40942-024-00580-2

**Published:** 2024-09-26

**Authors:** Rodrigo Anguita, Lorenzo Ferro Desideri, Anat Loewenstein, Martin Zinkernagel

**Affiliations:** 1 Department of Ophthalmology, Inselspital, University Hospital of Bern, Bern, Switzerland; 2 Moorfields Eye Hospital NHS Foundation Trust, City Road, London, EC1V 2PD UK; 3 Division of Ophthalmology, Tel Aviv Medical Center, Tel Aviv University, Tel Aviv, Israel

## Abstract

This editorial examines the transformative impact of the digital revolution on retinal practice, highlighting how technological advances are transforming patient care and pushing the boundaries of ophthalmology. It explores key areas of progress, including personalised medicine through big data, artificial intelligence and advanced imaging techniques; the role of telemedicine and home OCT in improving access and monitoring; advances in robotic surgery and 3D printing for vitreoretinal procedures; and the potential of large language models in patient education and communication. While highlighting the immense potential of these innovations, the editorial also addresses ethical considerations related to privacy and algorithmic bias. It emphasises the importance of multidisciplinary collaboration and maintaining a patient-centred approach in the digital age.

**Editorial**

In the constantly evolving healthcare system, we stand in the middle of a new era in retinal practice. The digital revolution has created extraordinary opportunities to improve patient care, outcomes and redefine the limits of what is possible in ophthalmology.

The convergence of big data, artificial intelligence (AI) and advanced imaging techniques has opened new frontiers in retinal care [1]. We are witnessing the beginning of personalised medicine, where treatments are tailored to individual, molecular, genetic profiles and clinical data. Predictive analytics now offer insights into disease progression and surgical outcomes, allowing clinicians to make more informed decisions. These advances not only improve patient care, but also facilitate research [2].

Telemedicine has emerged as a powerful tool in the management of retinal disease, breaking down geographical barriers and improving access to specialists [3]. Remote consultation, diagnosis and follow-up are now possible, ensuring that patients receive prompt consultation regardless of their location. This is particularly important for reaching those with less access and for screening for retinal disease. In addition, the recent introduction of home OCT is revolutionising treatment by allowing patients to be monitored from home, allowing early detection of changes, providing clinicians with more frequent data and improving the management of macular disease [4, 5].

Vitreoretinal surgery is also benefiting from technological advances. Robot-assisted surgery continues to improve and will certainly play an important role in procedures requiring extreme precision, such as cannulation of occluded retinal vessels [6]. Additionally, digital surgical techniques are increasing precision and expanding the capabilities of vitreoretinal surgeons. They are also improving the training of future ophthalmic surgeons. 3-D printing technologies are also an important part of the new technologies, creating customised ocular prosthetics, orbital implants and surgical models tailored to the individual patient’s anatomy. These innovations not only improve surgical outcomes, but also serve as powerful educational tools for the next generation of retinal specialists [7].

Large Language Models are playing an increasingly important role in ophthalmology, being tested as a tool to educate patients, assessing their performance in medical education and their ability to make diagnoses. There is no doubt that in the future, customised LLMs will have an important place in ophthalmology departments. Their potential applications include efficient patient triage, providing accurate responses to common patient inquiries, and enhancing overall patient communication [8, 9]. Their ability to process vast amounts of medical literature and adapt to new information makes them powerful tools for advancing ophthalmic care and improving patient outcomes.

As we move into the digital age, we must also consider the ethical implications and challenges that come with it. Data privacy, algorithmic bias and the digital divide are issues that demand our attention and thoughtful solutions. The way forward requires collaboration not only among ophthalmologists, but also with data scientists, engineers, ethics departments and regulatory bodies [10]. We must also remember that the patient is at the centre of all these technological advances and that quality is not only about improving outcomes but also about patient satisfaction and our ultimate goal remains the same: to preserve and restore sight and improve quality of life. The digital tools at our disposal are a way to achieve these goals, ensuring that they enhance, not replace, the human element of care, and that the patient’s experience and needs are always at the core of our practice.

This special issue serves as a platform for sharing cutting-edge research, innovative practices and visionary ideas. The future of retinal practice is being shaped now, and each of us has a role to play in helping to bring the power of digital technologies for the benefit of our patients.

The digital age in retina is not just about adopting new technologies; it is about reimagining what is possible in eye care and working tirelessly to make those possibilities a reality.

## Data Availability

No datasets were generated or analysed during the current study.
